# Human amniotic mesenchymal stem cells combined with PPCNg facilitate injured endometrial regeneration

**DOI:** 10.1186/s13287-021-02682-2

**Published:** 2022-01-12

**Authors:** Jiayue Huang, Wenwen Zhang, Jie Yu, Yating Gou, Nizhou Liu, Tingting Wang, Congcong Sun, Benyuan Wu, Changjiang Li, Xinpei Chen, Yanhua Mao, Yingfeng Zhang, Jia Wang

**Affiliations:** Department of Obstetrics and Gynecology, University-Town Hospital of Chongqing Medical University, No. 55, Daxuecheng Middle Road, Chongqing, 401331 China

**Keywords:** PPCNg, Human amniotic mesenchymal stem cells, Intrauterine adhesions, Endometrial regeneration

## Abstract

**Background:**

Caused by the injury to the endometrial basal layer, intrauterine adhesions (IUA) are characterized by uterine cavity obliteration, leading to impaired fertility. Human amniotic mesenchymal stem cells (hAMSCs) have the potential to promote endometrial regeneration mainly through paracrine ability. PPCNg is a thermoresponsive biomaterial consisted of Poly (polyethylene glycol citrate-co-*N*-isopropylacrylamide) (PPCN) mixed with gelatin, which has been reported as a scaffold for stem cell transplantation. This study aims to investigate the therapeutic effect of hAMSCs combined with PPCNg transplantation in promoting the regeneration of injured endometrium.

**Methods:**

hAMSCs were cultured in different concentrates of PPCNg in vitro, and their proliferation, apoptosis and cell cycle were examined by CCK-8 assay and flow cytometry. Immunofluorescence was used to determine the MSCs specific surface markers. The expression of pluripotent genes was analyzed by qRT-PCR. The multiple-lineage differentiation potential was further evaluated by detecting the differentiation-related genes using qRT-PCR and specific staining. The Sprague–Dawley (SD) rat IUA model was established with 95% ethanol. hAMSCs combined with PPCNg were transplanted through intrauterine injection. The retention of DiR-labeled hAMSCs was observed by vivo fluorescence imaging. The endometrium morphology was assessed using hematoxylin and eosin (H&E) and Masson staining. Immunohistochemistry staining was performed to detect biomarkers related to endometrial proliferation, re-epithelialization, angiogenesis and endometrial receptivity. The function of regenerated endometrium was evaluated by pregnancy tests.

**Results:**

hAMSCs maintained normal cell proliferation, apoptosis and cell cycle in PPCNg. Immunofluorescence and qRT-PCR showed that hAMSCs cultured in PPCNg and hAMSCs cultured alone expressed the same surface markers and pluripotent genes. hAMSCs exhibited normal multilineage differentiation potential in PPCNg. Vivo fluorescence imaging results revealed that the fluorescence intensity of hAMSCs combined with PPCNg intrauterine transplantation was stronger than that of direct hAMSCs intrauterine transplantation. Histological assays showed the increase in the thickness of endometrial and the number of endometrial glands, and the remarkably decrease in the fibrosis area in the PPCNg/hAMSCs group. The expressions of Ki-67, CK7, CK19, VEGF, ER and PR were significantly increased in the PPCNg/hAMSCs group. Moreover, the number of implanted embryos and pregnancy rate were significantly higher in the PPCNg/hAMSCs group than in the hAMSCs group.

**Conclusions:**

PPCNg is suitable for growth, phenotype maintenance and multilineage differentiation of hAMSCs. hAMSCs combined with PPCNg intrauterine transplantation can facilitate the regeneration of injured endometrium by improving utilization rates of hAMSCs, and eventually restore reproductive capacity.

**Supplementary Information:**

The online version contains supplementary material available at 10.1186/s13287-021-02682-2.

## Background

Intrauterine adhesions (IUA) are caused by damage to the endometrial basal layer, mainly resulting from invasive intrauterine operations and repeated endometrial infections [1], especially post-abortion/miscarriage curettage [2]. The main pathological feature of IUA is endometrial fibrosis, followed by the formation of adhesion bands, resulting in complete or partial uterine obliteration [3], causing a series of clinical symptoms such as reduced menstrual flow, amenorrhea, periodic abdominal pain, secondary infertility, recurrent abortion, premature delivery, and abnormal placental function, which seriously harm female reproductive health [4]. Transcervical resection of adhesions (TCRA) is the preferred treatment strategy for IUA [5]. However, the postoperative recurrence rate is high, especially for severe adhesion, which can be as high as 20–62.5% [4]. The pregnancy rate of patients who have suffered from severe IUA post-treatment is only up to 40.5% [6]. Currently, various therapies are adopted to prevent postoperative adhesion recurrence, including IUD, Foley balloon [3], estrogen [7], hyaluronic acid gel [8], and amniotic membrane transplantation [9]. Although these therapies can alleviate the recurrence of IUA to a certain degree, no significant improvements were found in the postoperative pregnancy rate. Thus, it is necessary to explore more effective therapeutic methods to repair the injured endometrium and improve the endometrial function.

Mesenchymal stem cells (MSCs) are known as a kind of adult stromal cells with self-renewal and multilineage differentiation potential [10]. Recent studies have shown that MSCs located at perivascular sites were transformed from pericytes. MSCs could rapidly respond to tissue damage, playing a role in the secretion of biological factors and immunomodulation, and promoted tissue repair synergistically with the immune systems [11–13]. MSCs transplantation has been extensively researched for tissue repair. It is reported that MSCs can secrete various growth factors and cytokines, such as VEGF, IGF-1 and IL-6, during tissue regeneration, thereby improving the somatic microenvironment, and regulating native stem cells proliferation, apoptosis, and differentiation processes [14–17]. Human amniotic mesenchymal stem cells (hAMSCs) are derived from discarded placentas, which are abundant in source with noninvasive acquisition and low-ethical controversy [18, 19]. In addition, hAMSCs have the advantages of low immunogenicity and no tumorigenicity, making them the ideal cell source for tissue repair [20, 21]. hAMSCs have been reported to enhance bone integration and bone regeneration [22]. Li et al. [21] found that hAMSCs can effectively heal skin damage induced by heat stress by promoting cell proliferation, inhibiting apoptosis of skin cells and accelerating epithelial regeneration. Gan et al. [23] transplanted hAMSCs by intramuscular injection and found that hAMSCs could regenerate the injured endometrium of IUA and regulate the expression of related inflammatory cytokines. However, the intrauterine colonization rate after MSCs transplantation was not stable. Cervello et al. [24] reported that CD133^+^ BMDSCs were recruited to the damaged horns after intrauterine injection or tail vein injection in the IUA murine model, accounting for 0.59% and 0.65% of the total uterine cells, respectively. Liu et al. [25] implanted GFP ^+^ cells into mice with mild endometrial injury by intravenous injection or intrauterine injection and found that the transplanted cells only accounted for 0.02–0.5% of the total number of uterine cells. Therefore, improving cell survival ability and utilization rate is the key to strengthen stem cell-based endometrial repair.

Hydrogel scaffolds are a kind of biomaterials providing a place for cell growth and attachment to improve the colonization rate of cells at the transplanted site [26]. Poly (polyethylene glycol citrate-co-*N*-isopropylacrylamide) (PPCN) is a thermoresponsive biomaterial which undergoes the reversible phase change from liquid to solid at 37 °C [27]. PPCN has been used as a carrier which sustainedly releasing bioactive SDF-1 after transplantation to promote dermal wounds closure without causing obvious inflammatory response [28]. By adding gelatin, PPCNg is formed to increase cell adhesion and support cell survival and implantation [29]. With biocompatibility, PPCNg has been reported to mediate BMP9-transduced MSCs, including iMEFs [29], iMADs [30] and iCALs [31], which were located at bone defect sites to enhance bone formation and angiogenesis. A recent study has shown that PPCNg combined with hSMSCs transplantation can effectively promote chondrogenic differentiation of hSMSCs by improving nutrient supply at the injured site and providing regular space for cell growth [32].

This study investigated whether hAMSCs combined with PPCNg intrauterine transplantation could promote the regeneration of injured endometrium in IUA. The results showed that hAMSCs proliferated well, and maintained stemness and differentiation potential when cultured in PPCNg. Moreover, hAMSCs combined with PPCNg intrauterine transplantation could significantly enhance the cell colonization and utilization rate, promote the recovery of endometrial structure and function, and effectively restore reproductive function.

## Methods

### Synthesis of PPCNg

The PPCNg was synthesized according to reported method [27, 29]. PPCN powder (Molecular Oncology Laboratory, University of Chicago Medical Center) was dissolved in phosphate-buffered saline (PBS), prepared into different concentrations of stock solution (60 mg/mL, 80 mg/mL, 100 mg/mL, 120 mg/mL), filtered with 0.22-μm filters for sterilization, and stored at 4 °C. Then, PPCN-gel (PPCNg) was formed by mixing different concentrations of PPCN stock solution with 0.2% gelatin/PBS (Sigma) in a 1:1 ratio, and the final concentrations of PPCN were 30 mg/mL, 40 mg/mL, 50 mg/mL, 60 mg/mL, respectively.

### hAMSCs proliferation, apoptosis and cell cycle in vitro

Cell counting Kit-8 (CCK-8) (Dojindo) was used to assess the proliferation. hAMSCs suspension was seeded in a 96-well plate (5000 cells/100μL/well). Cells were cultured in PPCNg for 3 days, then the medium was replaced with a medium containing 10 μL CCK-8 (100 μL/well) and incubated at 37 °C for 1 h. The absorbance was determined at the wavelength of 450 nm using an automatic enzyme plate analyzer (Bio-Rad).

hAMSCs were digested and collected after being cultured in PPCNg for 3 days and incubated with propidium iodide (Beyotime) at 4 °C for 30 min in the dark. Flow cytometry was used to analyze the cell cycle.

hAMSCs were cultured in PPCNg for 3 days, and then cell apoptosis was detected by Annexin V-FITC Apoptosis Detection Kit (Solarbio); 100 μL cells (1 × 10^5^ cells) were incubated with 5 μL Annexin V-FITC for 10 min and then incubated with 5 μL PI for 5 min in the dark. The cells were detected by flow cytometry within 1 h.

### Immunofluorescence staining

hAMSCs were fixed with 4% paraformaldehyde (Biosharp, China) for 20 min, and with permeabilized 0.1% Triton-X 100 (Sigma) for 15 min and were blocked with 5% bovine serum albumin (BSA) (Bosterbio) for 30 min at room temperature. The cells were incubated overnight at 4 °C with the primary antibodies anti-Integrin beta 1 (CD29) (ab134179, Abcam), anti-CD44 (ab189524, Abcam), anti-CD73 (ab133582, Abcam), anti-CD105 (ab231774, Abcam), anti-CD19 (ab134114, Abcam), anti-CD34 (ab81289, Abcam), anti-CD45 (ab40763, Abcam), anti-cytokeratin 7 (CK7) (ab181598, Abcam) and anti-cytokeratin 19 (CK19) (ab52625, Abcam) and then incubated with the Alexa Fluor 488-conjugated secondary antibody (ab150077, Abcam) for 1 h at 37 °C in the dark. The cell nuclei were stained with 4′,6-diamidino-2-phenylindole (DAPI) (Sigma) for 5 min. The results were observed by fluorescence microscope.

### Quantitative real-time polymerase chain reaction (qRT-PCR)

Total RNA was isolated with RNAiso Plus reagent kit (Takara, Japan) according to the manufacturer's instructions. cDNA was generated by reverse transcription of RNA using PrimeScript RT reagent kit (Takara, Japan) following the manufacturer’s guidelines. The PCR reaction system was performed with specific primers and SYBR Premix Ex Taq II (Takara, Japan), with a final volume of 20 μL. The PCR cycling conditions were as follows: pre-denaturation at 95 °C for 30 s, denaturation at 95 ˚C for 5 s, annealing at 60 °C for 30 s, 40 cycles, and elongation at 60 °C for 30 s. Each sample was assayed in triplicate. Primer sequences are listed in Table 1. Expression of target genes was normalized to glyceraldehyde phosphate dehydrogenase (GAPDH). The relative genes expression levels were calculated using the 2^−△△Ct^ method.

**Table 1:** Primer sequence of genes used in qRT-PCR analyses

Gene	Forward primer sequence (5′-3′)	Reward primer sequence (5′-3′)
OCT4	GCAAGCCCTCATTTCACC	CCATCACCTCCACCACCT
SOX2	CGAACCATCTCTGTGGTCT	GTGTCAACCTGCATGGC
KLF4	AGGAGCCCAGCCAGAAA	TCCAGTCACAGACCCCATC
C-MYC	GCCAGAGGAGGAACGAG	GCTTGGACGGACAGGAT
CK7	TGGATGCCCTGAATGATGAGAT	GGGAGCGACTGTTGTCCA
CK8	CCATTAAGGATGCCAACGCCAA	TTCATCAGCTCCTGGTACTCAC
CK19	ACTACACGACCATCCAGGAC	GCAGAGCCTGTTCCGTCTCA
E-CDA	ACCATTCAGTACAACGACCCAA	GCCTTCCTACAGACGCCAG
ALP	GTACCATTTGCCCAGGTG	GGGCTCGCTCATCAAAG
RUNX2	GTAGATGGACCTCGGGAAC	TGCGCTACCTGAAACTGA
BSP	GGAGGAGACAATGGAGA	TTCAACGGTGGTGGTTTT
COL-II	GGATGGCTGCACGAAAC	CCCTATGTCCACACCGAAT
ACAN	TGCAGAACAGTGCCATCA	CTCCATAGCAGCCTTCCC
SOX9	AGCGCATTACCCACTTGT	ATCCTCCACGCTTGCTC
PPARγ	CGAGAAGGAGAAGCTGTTG	TCAGCGGGAAGGACTTTA
ADIP	TATCCCCAAGCCACACC	TCAGCCAGAGCAATGAGAT
LPL	TGCGTCTCTTTTGTTCCTG	CTCCATCTGGCTCATTGC
GAPDH	AACAGCCTCAAGATCATCAGC	ATGAGTCCTTCCACGATACCAA

OCT4, octamer-binding transcription factor-4; SOX2, SRY-related high-mobility-group-box protein-2; KLF4, Kruppel-like factor-4; C-MYC, cellular-myelocytomatosis viral oncogene; CK-7, cytokeratin 7; CK-8, cytokeratin 8; CK-19, cytokeratin 19; E-CDA, E-Cadherin; ALP, alkaline phosphatase; RUNX2, runt-related transcription factor-2; BSP, bone sialoprotein; SOX9, SRY-BOX Transcription Factor 9; ACAN, aggrecan; COL-II, type II collagen; PPARγ peroxisome proliferator-activated receptor γ; LPL, lipoprotein lipase; ADIP, adiponectin; GAPDH, glyceraldehyde-3-phosphate dehydrogenase

### Multilineage differentiation of hAMSCs in vitro

For osteogenesis, hAMSCs were cultured in an induction medium with the addition of 10% FBS, 10 mM sodium β-glycerol phosphate (Sigma), 100 nM dexamethasone (Sigma), and 0.05 mM ascorbic acid (Solarbio) for 21 days, and then determined by Alizarin Red S staining to examine calcium deposits. The cells were fixed with 4% paraformaldehyde for 20 min, and were incubated with Alizarin Red S solution (Solarbio) for 30 min and washed thoroughly. The staining cells were observed under an inverted microscope.

For adipogenesis, hAMSCs were cultured in an induction medium supplemented with 10% FBS, 0.5 mM isobutylmethylxanthine (IBMX) (Sigma), 1 mM dexamethasone (Sigma), 100 mM indomethacin (Solarbio), and 10 mg/L insulin for 14 days, and detected by Oil Red O staining to observe lipid droplets. The cells were fixed with ORO fixative for 20 min, soaked in 60% isopropanol for 5 min, incubated in ORO stain (Solarbio) at room temperature for 20 min, and repeatedly washed to remove unbound dyes. The staining cells were observed under an inverted microscope.

For chondrogenesis, hAMSCs were cultured in an induction medium composed of 10 ng/mL TGF-β1 (PeproTech), 100 nM dexamethasone (Sigma), 50 μg/mL ascorbic acid (Solarbio), and 40 μg/mL L-proline (Solarbio) for 21 days. Toluidine blue staining was performed to observe the chondrogenic differentiation. The cells were fixed with 4% paraformaldehyde for 20 min, stained with toluidine blue solution (Solarbio) for 5 min, and washed extremely to remove unbound dyes. The staining cells were observed under an inverted microscope.

For endometrial differentiation, when hAMSCs reached 30–40% confluence, the medium was exchanged for DMEM/F12 supplemented with 2%FBS, 10 ng/mL TGF-β1 (PeproTech), 10 ng/mL EGF (PeproTech), 10 ng/mL PDGF-BB (PeproTech), 1 × 10^–6^ mol/L β-estradiol (Solarbio), and endometrial conditioned medium. Uninduced cells were cultured with DMEM/F12 containing 2% FBS. After 3 days, the expression of CK7 and CK19 was detected by immunofluorescence.

Multilineage differentiation was also tested by qRT-PCT. hAMSCs were infected with Ad-BMP9 or Ad-RFP, and 1 μL/mL Polymix (Denovo) was added to improve the infection efficiency. At 72 h after infection, more than 80% of the cells were infected. After 5 days of culture, qRT-PCT was performed to analyze the expression of multilineage differentiation-related genes. Primer sequences are shown in Table 1.

### Establishment of the IUA model

Animal experiment protocols were approved by the Ethics Committee of Chongqing Medical University (No. 2014050). Female Sprague–Dawley (SD) rats, weighing 200–230 g, were purchased from the Animal Experimental Center of Chongqing Medical University, raised in a controlled environment at 22ºC with a 12 h/12 h light/dark cycle. Rats in diestrus were selected based on the vaginal smear analysis, and anesthetized with 5% chloral hydrate anesthesia (Biosharp) (10 ml/kg) by intraperitoneal injection, and the skin and muscle were cut midline in the low abdomen to expose the uterus. The two ends of the right uterine horns were gently clamped with toothless tweezers, 95% ethanol was injected until the uterine cavity was filled and maintained for 3 min, and the uterine cavities were rinsed with saline. The left uterine horns were untouched. Then, the uterus was put back to the normal position, and the abdominal incision was sutured.

### Transplantation of hAMSCs

A total of 80 rats were randomly assigned to 5 groups: the sham-operated group, the IUA group, the PPCNg group, the hAMSCs group, and the PPCNg/hAMSCs group (*n* = 16 in each group). Two weeks after the establishment of IUA model, different treatment measures were performed in each group. In the sham-operated group, the right uterine cavity had saline injected only instead of 95% ethanol. In the IUA group, no treatment was given. In the PPCNg group, 100 μL PPCNg was injected into the right uterine cavity. In the hAMSCs group, hAMSCs were transplanted directly by intrauterine injection. 1 × 10^7^ hAMSCs were resuspended in 100 μL PBS, and then injected into the right uterine cavity. In the PPCNg/hAMSCs group, hAMSCs were combined with PPCNg for intrauterine injection transplantation. 1 × 10^7^ hAMSCs were resuspended in 100 μL PPCNg, kept on ice to prevent premature solidification of material, and subsequently injected into the right uterine cavity.

### In vivo fluorescence imaging

hAMSCs were labeled with DiR (AAT Bio, USA) before intrauterine transplantation. 1 × 10^6^ cells were incubated in 5 μM DiR working solution at 37 °C for 20 min, washed twice with serum-free DMEM/F12, and cultured in a medium containing 10% FBS. The rats were subjected to imaging with in vivo imaging system (LB983, Berthold, Germany). The fluorescence images were obtained at 1d, 3d, 7d after transplantation. Quantitative analysis was done with image software (IndiGo).

### Histological analysis

Two weeks after transplantation, the rats were sacrificed for harvesting uterine specimens which were fixed in 4% paraformaldehyde, dehydrated, embedded in paraffin, and sliced into sections. H&E staining and Masson staining were performed according to the manufacturer’s instructions (Solarbio). H&E staining was used to measure the endometrial thickness and to count the gland numbers. Masson staining was conducted to calculate the percentage of fibrosis area (endometrial fibrosis areas/total endometrial areas). The collagen fibers appeared blue under the microscope. Image J software was applied to analyze the images.

### Immunohistochemistry analysis

The primary antibodies used were anti-CK7 (ab181598, Abcam), anti-CK19 (ab52625, Abcam), anti-Ki67 (ab16667, Abcam), anti-vascular endothelial growth factor (VEGF) (ab52917, Abcam), anti-estrogen receptor (ERα) (ab16660, Abcam), and anti-progesterone receptor (PR) (ab16661, Abcam). The paraffin sections were deparaffinized and hydrated. The antigen was retrieved with EDTA buffer. The nonspecific antibody binding sites were blocked by goat serum. The sections were incubated at 4 °C overnight with primary antibodies and subsequently incubated with biotin-labeled secondary antibody at 37 °C for 1 h. The color reaction was developed with diaminobenzidine (DAB) (ZSGB Bio) and nuclei were stained with hematoxylin (Solarbio). The positive staining cells were observed as brownish-yellow under the microscope. Image J was applied to measure the percentage of positive staining area.

### Fertility test

Four weeks after transplantation, female rats were mated at a 1:2 ratio with sexually mature male rats. The day of vaginal plug presence in female rats was regarded as gestation day 0. At gestation day 14, the rats were sacrificed to collect the uterus, and the number of implanted embryos was counted.

### Statistical analysis

Statistical analysis was performed with SPSS version 22.0. The data were presented as mean ± standard deviation from at least three independent experiments. Two-group comparisons were performed using a t test and multiple-group comparisons were determined by one-way analysis of variance (ANOVA). *P* < 0.05 was considered statistically significant. (*indicate *P* < 0.05 and ** indicate *P* < 0.01).

## Results

### Characteristics of PPCNg

**Fig. 1:**
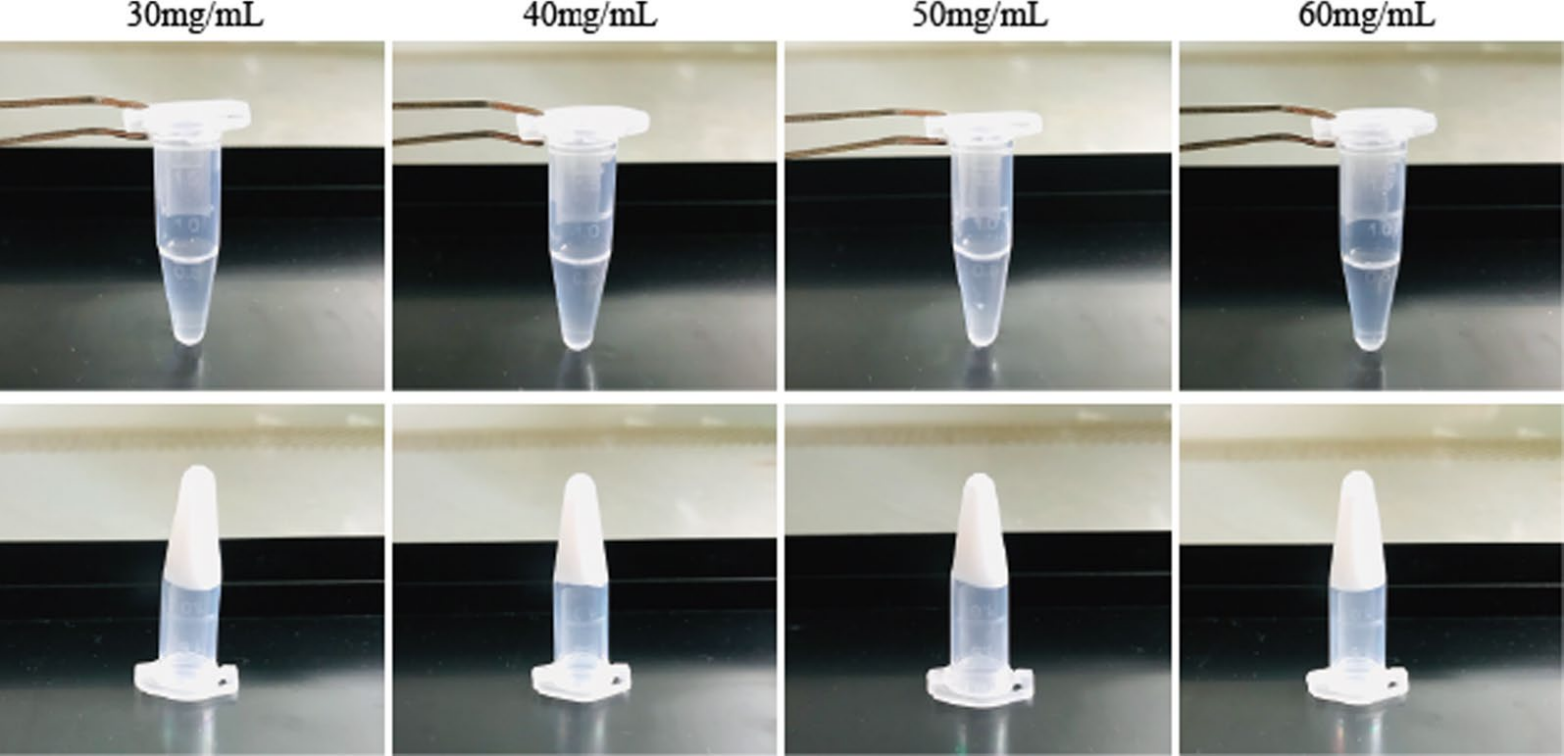
Physical appearance of PPCNg. PPCNg showed liquid at low temperatures and formed gel at 37 °C

The physical appearance of PPCNg with different concentrations (30 mg/mL, 40 mg/mL, 50 mg/mL, 60 mg/mL) remained liquid at 4 °C and formed uniform gel rapidly at 37 °C without significant alteration in volume (Fig. 1).

### Effects of PPCNg on hAMSCs proliferation, apoptosis and cell cycle in vitro

**Fig. 2:**
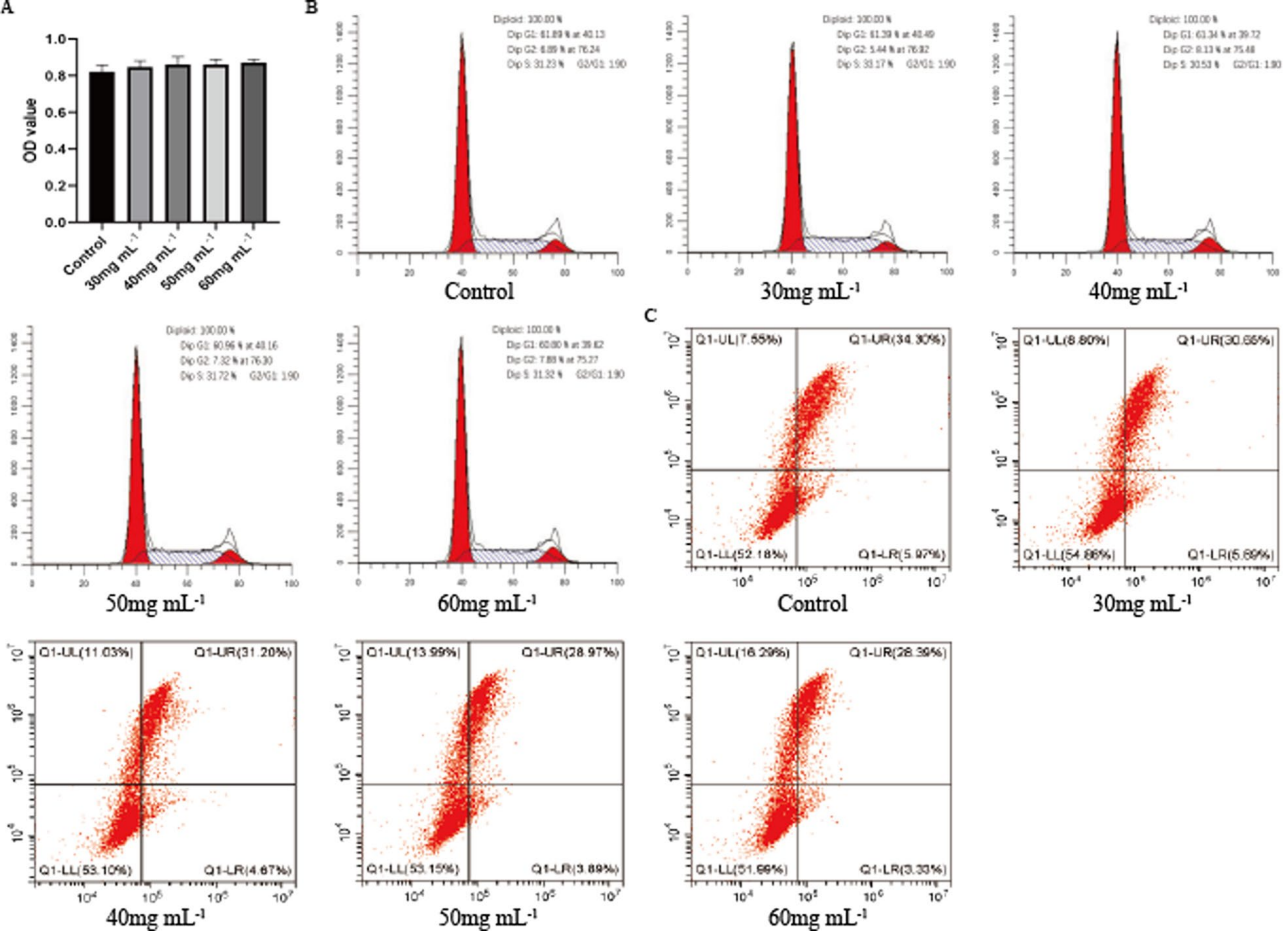
The proliferation, apoptosis and cell cycle of hAMSCs in different concentrations of PPCNg. **A** The results of CCK-8 assay. **B** The results of apoptosis by flow cytometry. **C** The results of cell cycle by flow cytometry

The isolated hAMSCs were identified by detecting specific surface markers (Additional file 1: Fig. S1). hAMSCs grew well when cultured in 30 mg/mL PPCNg (P1 group), 40 mg/mL PPCNg (P2 group), 50 mg/mL PPCNg (P3 group) and 60 mg/mL PPCNg (P4 group). After 3 days of culture in PPCNg, the proliferation ability of hAMSCs was detected by CCK-8 assay, and the results showed no significant difference between P1, P2, P3, P4 group and the control group (Fig. 2A). In addition, cell cycle analysis examined by flow cytometry demonstrated that the percent of cells in G2/M + S phase in P1, P2, P3 and P4 group had no significant difference compared with the control group (Fig. 2B). The results of the apoptosis test showed no statistical difference in apoptosis rate between P1, P2, P3, P4 group and the control group (Fig. 2C). The above results suggested that PPCNg at different concentrations had no toxic effect on hAMSCs. PPCNg at a concentration of 50 mg/mL was selected for the following studies.

### Effects of PPCNg on hAMSCs phenotype and stemness in vitro

**Fig. 3:**
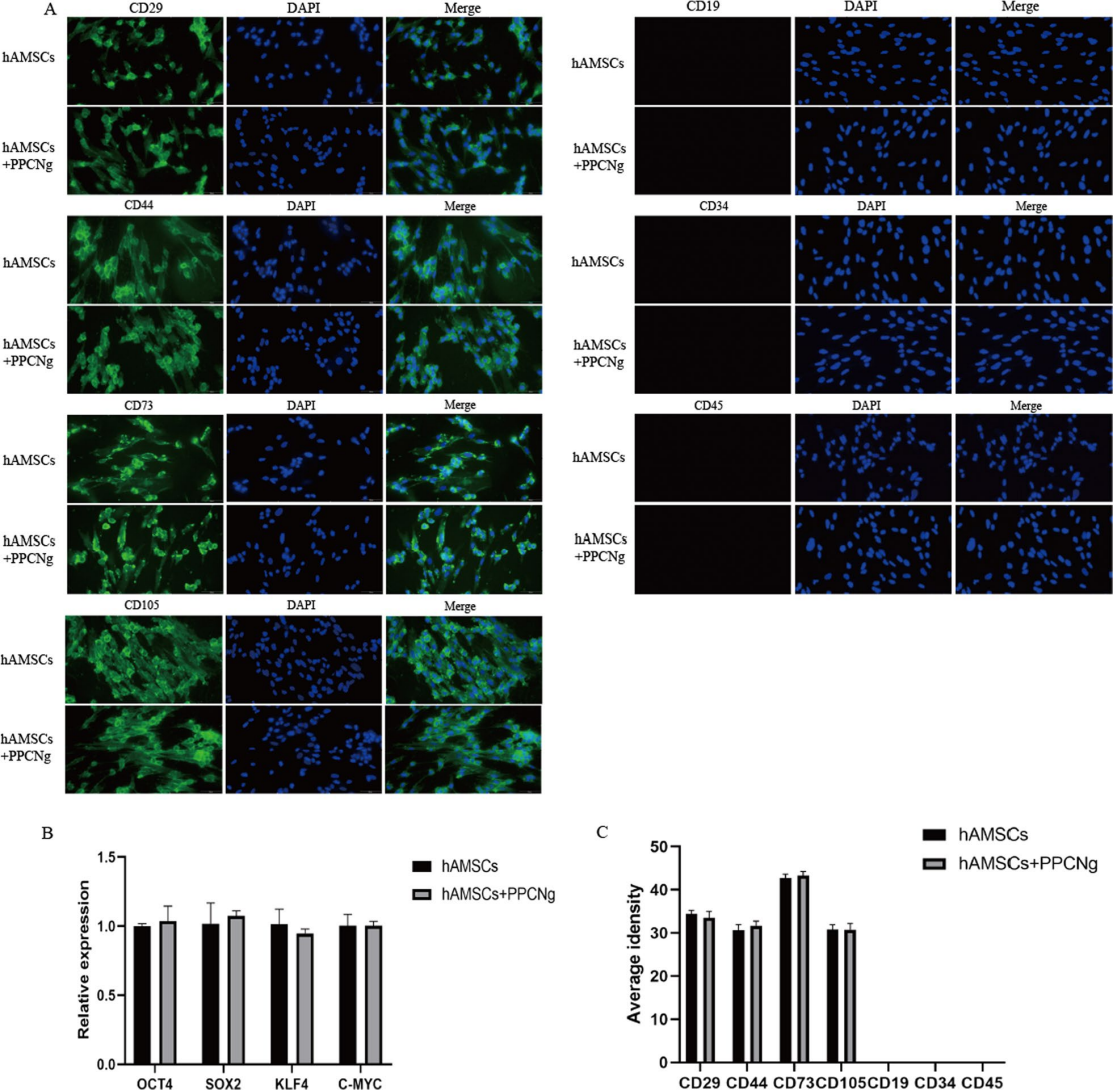
Phenotype and stemness of hAMSCs. **A** Immunofluorescence staining of MSCs specific surface markers. **B** Relative expression of OCT4, SOX2, KLF4 AND C-MYC. **C** Statistical analysis of the average density. (*n* = 3)

Phenotype is one of the key characteristics of MSCs. Immunofluorescence staining was conducted to examine the mesenchymal stem cell markers of hAMSCs, cultured in PPCNg for 3 days. The results showed that hAMSCs in PPCNg positively expressed CD29, CD44, CD73 and CD105, and negatively expressed CD19, CD34 and CD45. No significant difference was found in the expression levels of surface markers between the cocultured hAMSCs and the culture-isolated hAMSCs (Fig. 3A). Pluripotency genes, such as OCT4, SOX2, KLF4 and C-MYC, are known to be essential for maintaining self-renewal and differentiation abilities [33, 34]. To confirm the effect of PPCNg on the stem cell properties of hAMSCs, the mRNA expressions of OCT4, SOX2, KLF4 and C-MYC were assessed. The qRT-PCR results showed no statistical difference in mRNA expression of pluripotency genes between cocultured hAMSCs and culture-isolated hAMSCs indicating that PPCNg had no impact on the stemness of hAMSCs (Fig. 3B).

### Multilineage differentiation of hAMSCs in PPCNg

**Fig. 4:**
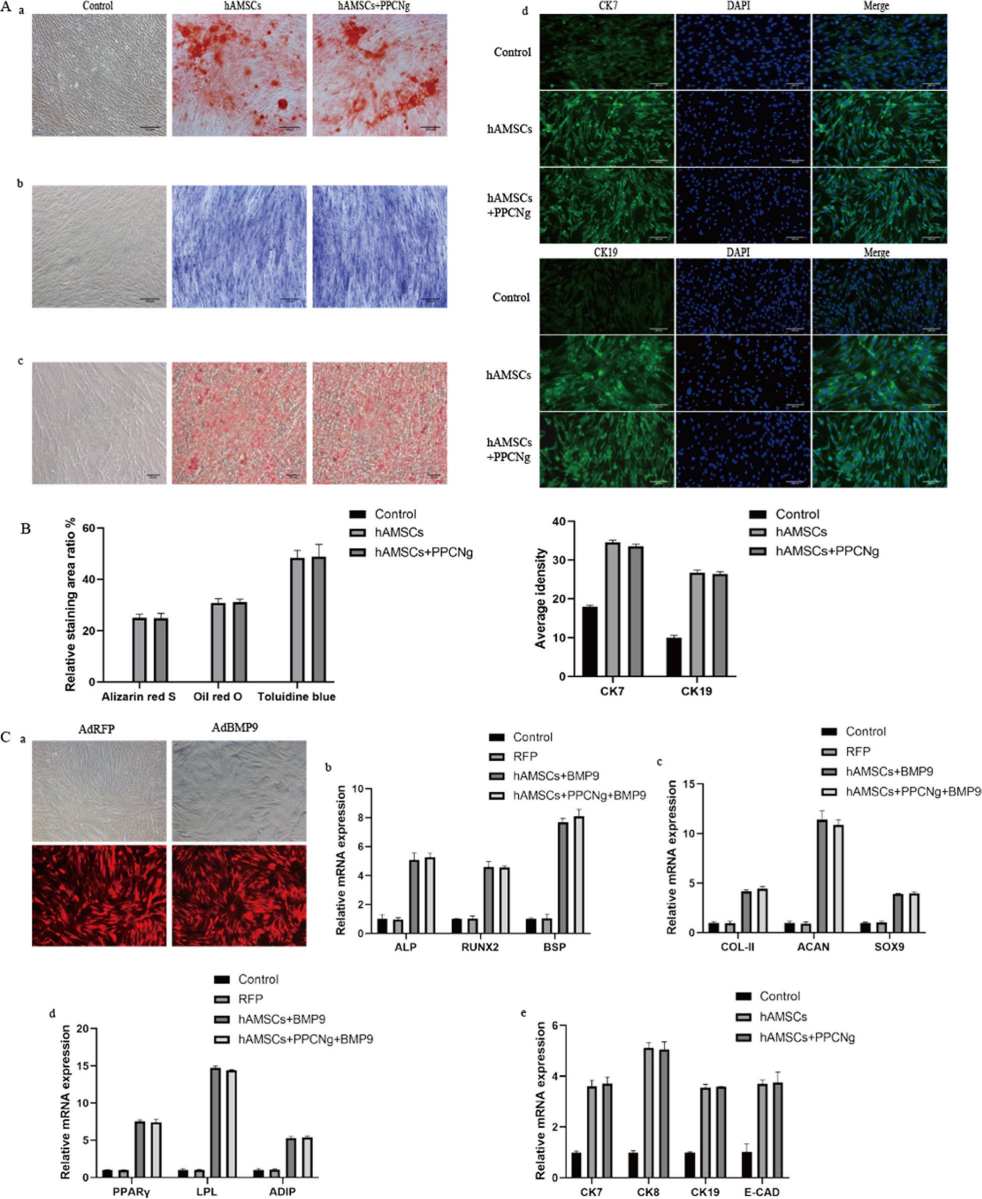
Multilineage differentiation potential of hAMSCs. **A** The induced hAMSCs were determined by (a) Alizarin red S staining, (b) Toluidine Blue staining, (c) Oil Red O staining, (d) immunofluorescence staining of CK7, CK19. **B**) Statistical analysis of the percentage of positive staining area and average density. **C** Relative expression of differentiation-related genes. (a) hAMSCs were infected by adenovirus. Relative expression of (b) ALP, RUNX2, BSP, (c) COL-II, ACAN, SOX9, (d) PPARγ, LPL, ADIP, (e) CK7, CK8, CK18 and E-CAD. (*n* = 3)

To assess the effect of PPCNg on the multilineage differentiation ability of hAMSCs, cells were induced for endometrial, osteogenic, chondrogenic and adipogenic differentiation. The differentiation potential was evaluated by specific staining tests. Twenty-one days post-osteogenic induction, the calcium salt deposition was observed by Alizarin red S staining, in which the mineralized matrix and nodules were shown red. The staining degree was similar between the coculture group and the isolated-culture group, suggesting no statistical difference in the amount of calcium deposition. Twenty-one days post-chondrogenic induction, Toluidine Blue staining was performed to identify the chondrocytes in which the extracellular matrix (ECM) was stained blue-purple, without significant difference in the degree of staining between the coculture group and the isolated-culture group. Fourteen days post-adipogenic induction, lipid vacuoles were visible in the cytoplasm, and the lipid droplets were stained red with Oil Red O staining, which showed no statistical difference in the number of lipid droplets between the coculture group and the isolated-culture group. Three days after the induction of endometrial differentiation, the expression of CK7 and CK19 was increased with no significant difference between the coculture group and the isolated-culture group. CK7 and CK19 were weakly expressed in the non-induced group (control group) (Fig. 4A).

The effect of PPCNg on differentiation-related genes of hAMSCs was further analyzed. BMP9 can induce osteogenic, chondrogenic, and adipogenic lineage-specific differentiation of mesenchymal stem cells [35–37]. hAMSCs were infected with either AdBMP9 or AdRFP. At 5d after infection, the fluorescence intensity and distribution were observed under the microscope. qRT-PCR was used to detect the expression of osteogenesis-related markers of ALP, RUNX2, and BSP, chondrogenesis-related markers of SOX9, ACAN, and COL-II, and adipogenesis-related markers of PPARγ, LPL, and ADIP. The results revealed that the expression levels of all marker genes in the infected groups were significantly upregulated, with no statistical difference between the coculture group and the isolated-culture group. At 3d after endometrial differentiation induction, the mRNA expression of epithelial markers, including CK7, CK8, CK19 and E-cadherin, was remarkably increased in induced group. There was no significant difference in the expression of CK7, CK8, CK19 and E-cadherin between the coculture group and the isolated-culture group **(**Fig. 4C**)**.

### Colonization of hAMSCs combined with PPCNg transplantation

**Fig. 5:**
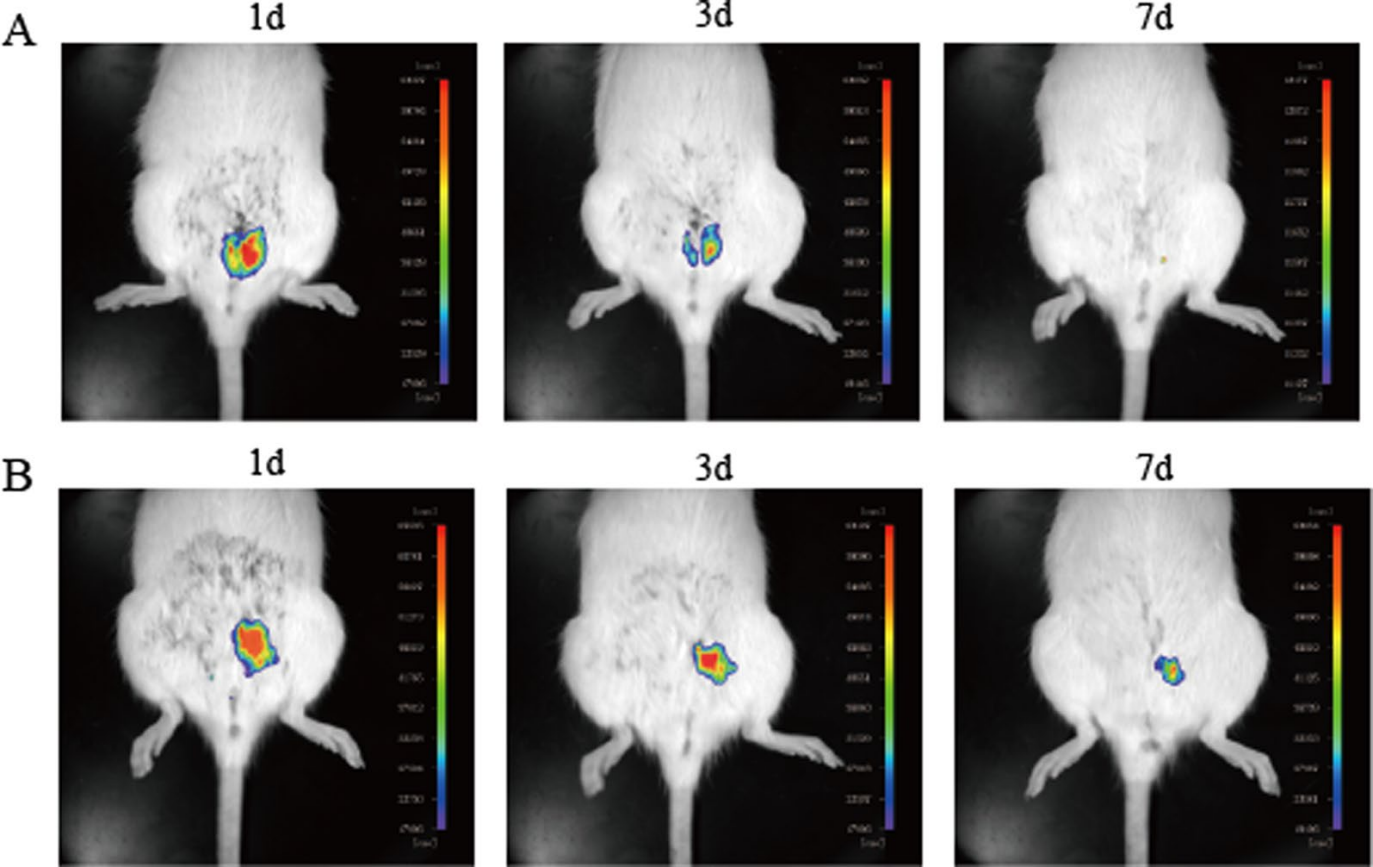
The colonization of hAMSCs in vivo at 1d, 3d and 7d. **A** hAMSCs group. **B** PPCNg/hAMSCs group

Before transplantation, hAMSCs were labeled with cell-tracer DiR. After intrauterine injection of hAMSCs and PPCNg/hAMSCs, in vivo fluorescence imaging was used to observe the colonization of the engrafted hAMSCs in utero at 1d, 3d and 7d. On the 1d after transplantation, the intensity of fluorescence was shown almost similar in the uterine cavity of the modeling side in both groups. On the 3d after transplantation, the fluorescence in both groups decreased, but the fluorescence intensity in the PPCNg/hAMSCs group was significantly higher than that in the hAMSCs group. Moreover, engrafted cells in the hAMSCs group were examined to spread to the vagina or abdominal cavity. With the extension of time, the fluorescence intensity in both groups further decreased to varying degrees on the 7d after transplantation. Energetic fluorescence was still detected in the PPCNg/hAMSCs group, while the fluorescence in the hAMSCs group reduced rapidly and could hardly be observed. The weakening rate of fluorescence increased with the extension of treatment time (Fig. 5). The results suggested that PPCNg could improve the colonization rate of hAMSCs in the uterine cavity to promote the hAMSCs utilization rate.

### Recovery of endometrium morphology

Two weeks after treatment, uterine tissues were collected for histological analysis. The morphology of the endometrium was observed by H&E staining and Masson staining. In the Sham group, the uterine cavity was open, the endometrium structure was intact with columnar epithelium on the surface, the abundance of glands mainly distributed in the submucosa and basal layer, and the collagen fibers appeared light blue. In the IUA group, the cavity was constricted or atresia, the endometrium became thinner, the glands and blood vessels were strongly reduced, and a large number of dark blue collagen fibers were seen.

**Fig. 6:**
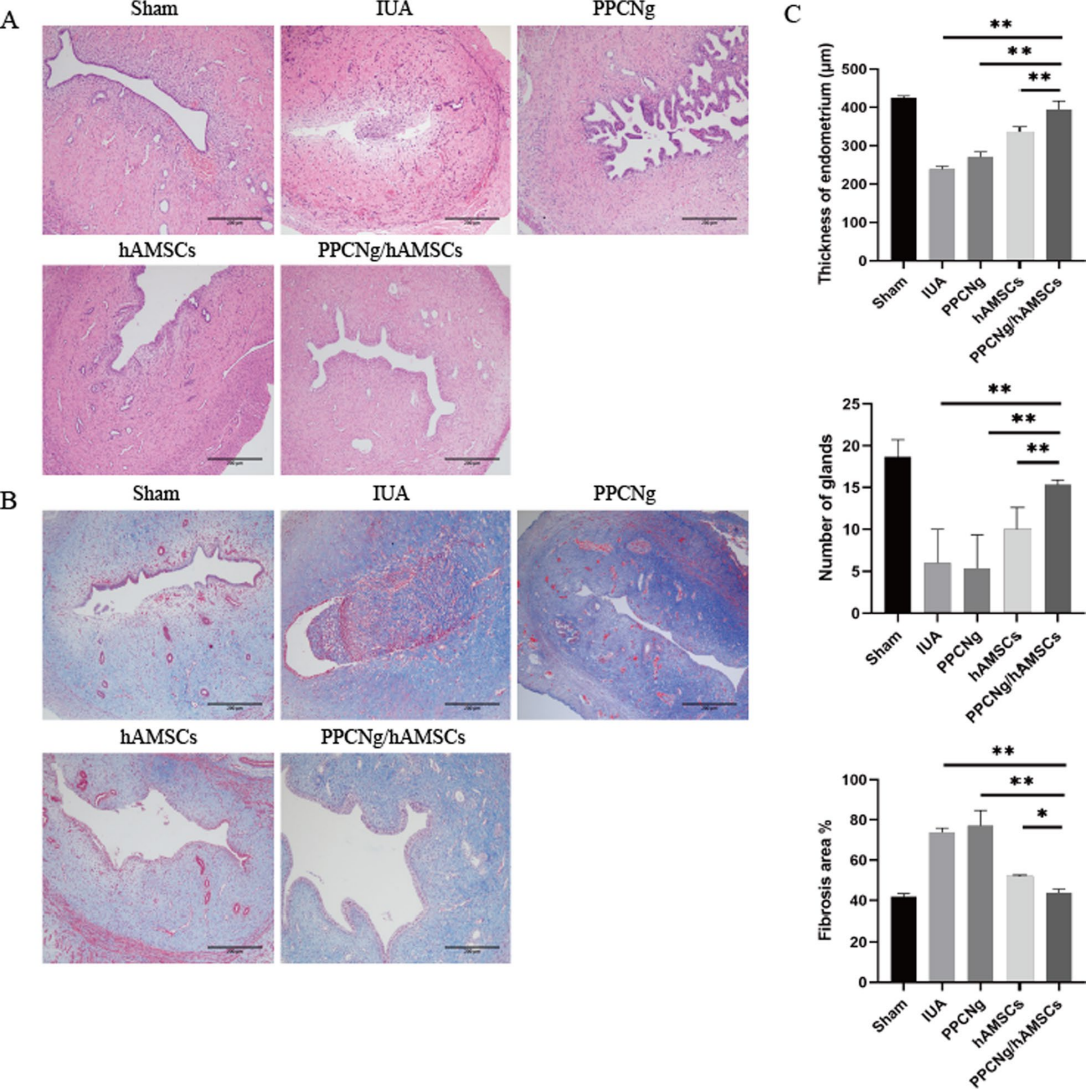
Morphological features of uteri. **A** H&E staining of uterine tissue (Scale bar = 200 μm)). **B** Masson staining of uterine tissue (Scale bar = 200 μm). **C** Statistical analysis of the endometrial thickness, number of glands and percentage of fibrosis area in each group. (*n* = 3, *P < 0.05, **P < 0.01)

The endometrial was thickest in the Sham group (425.24 ± 4.71) among the five groups. The endometrial thickness in the PPCNg/hAMSCs group (394.80 ± 21.49) was significantly higher than that in the hAMSCs group (336.87 ± 12.30) (*P* < 0.01) and the PPCNg group (263.23 ± 27.46) (*P* < 0.01). Similarly, the number of glands was significantly higher in the PPCNg/hAMSCs group (14.00 ± 1.73) than that in the hAMSCs group (12.66 ± 1.05) (*P* < 0.05) and the PPCNg group (5.33 ± 4.04) (*P* < 0.01) (Fig. 6A). Masson staining showed that the percentage of fibrosis area in the PPCNg/hAMSCs group (45.6 ± 0.4%) was similar to that in the Sham group (45.5 ± 5.9%) (*P* > 0.05), but significantly lower than that in the hAMSCs group (55.4 ± 4.5%) (*P* < 0.01), the PPCNg group (75.1 ± 4.8%) (*P* < 0.01) and the IUA group (76.3 ± 4.4%) (*P* < 0.01). The results revealed that PPCNg/hAMSCs could promote the morphological recovery of endometrium and reduce fibrosis formation (Fig. 6B).

### Regeneration of endometrium

**Fig. 7:**
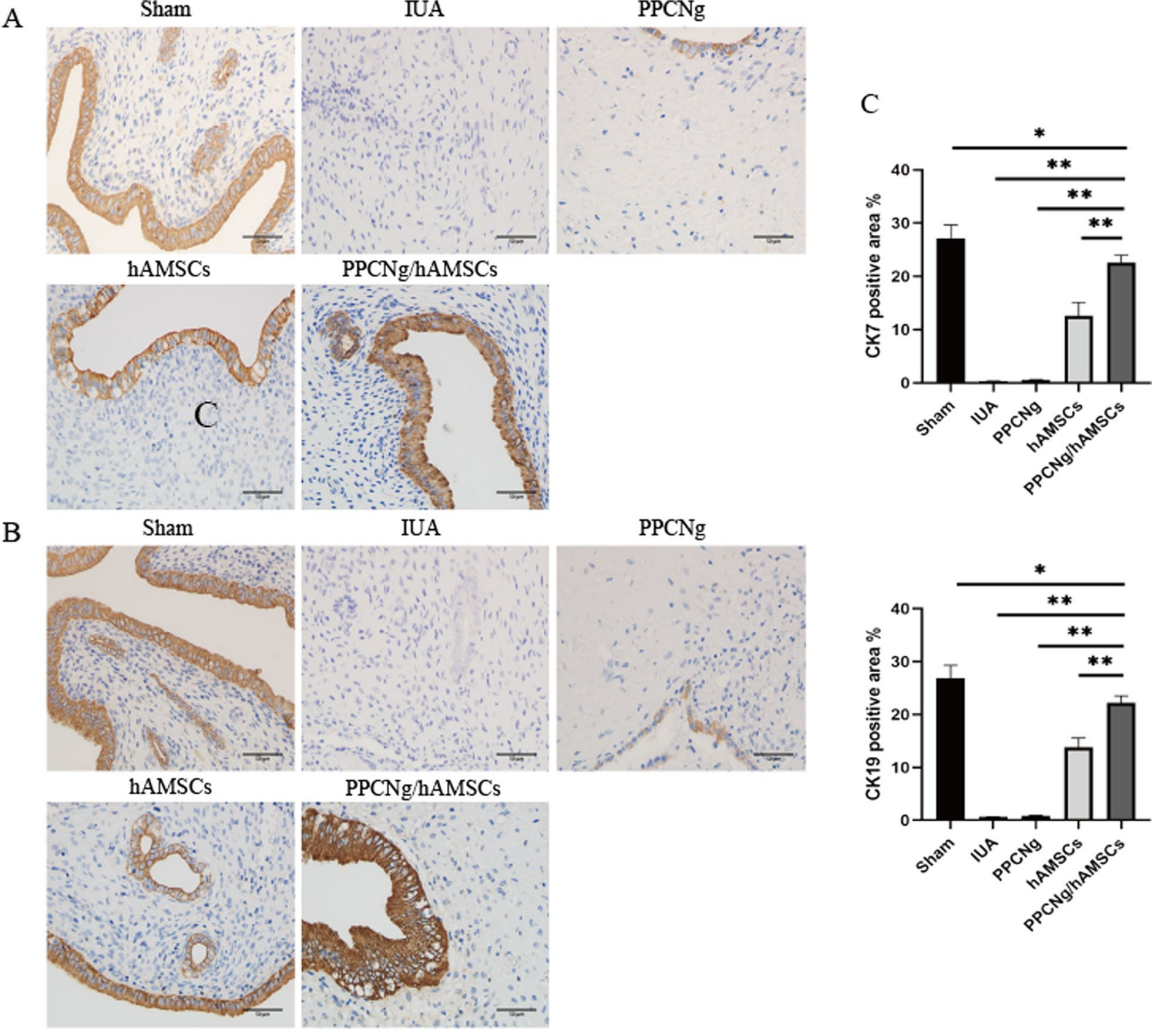
Endometrial epithelial recovery. **A** Immunohistochemical staining of CK7 (Scale bar = 50 μm). **B** Immunohistochemical staining of CK19 (Scale bar = 50 μm). **C** Statistical analysis of the percentage of CK7, CK19 positive areas. (*n* = 3, *P < 0.05, **P < 0.01)

CK7 and CK19 are cytoskeleton proteins that maintain the integrity of epithelial cells, which are expressed in the cytoplasm of endometrial epithelial cells. The expressions of CK7 and CK19 in the PPCNg/hAMSCs group (21.87 ± 2.13%, 22.88 ± 2.37%) were significantly higher than those in the hAMSCs group (12.58 ± 2.54%, 13.77 ± 1.78%) (*P* < 0.01) and the PPCNg group (0.57 ± 0.07%, 0.83 ± 0.07%) (*P* < 0.01) (Fig. 7**)**.

**Fig. 8:**
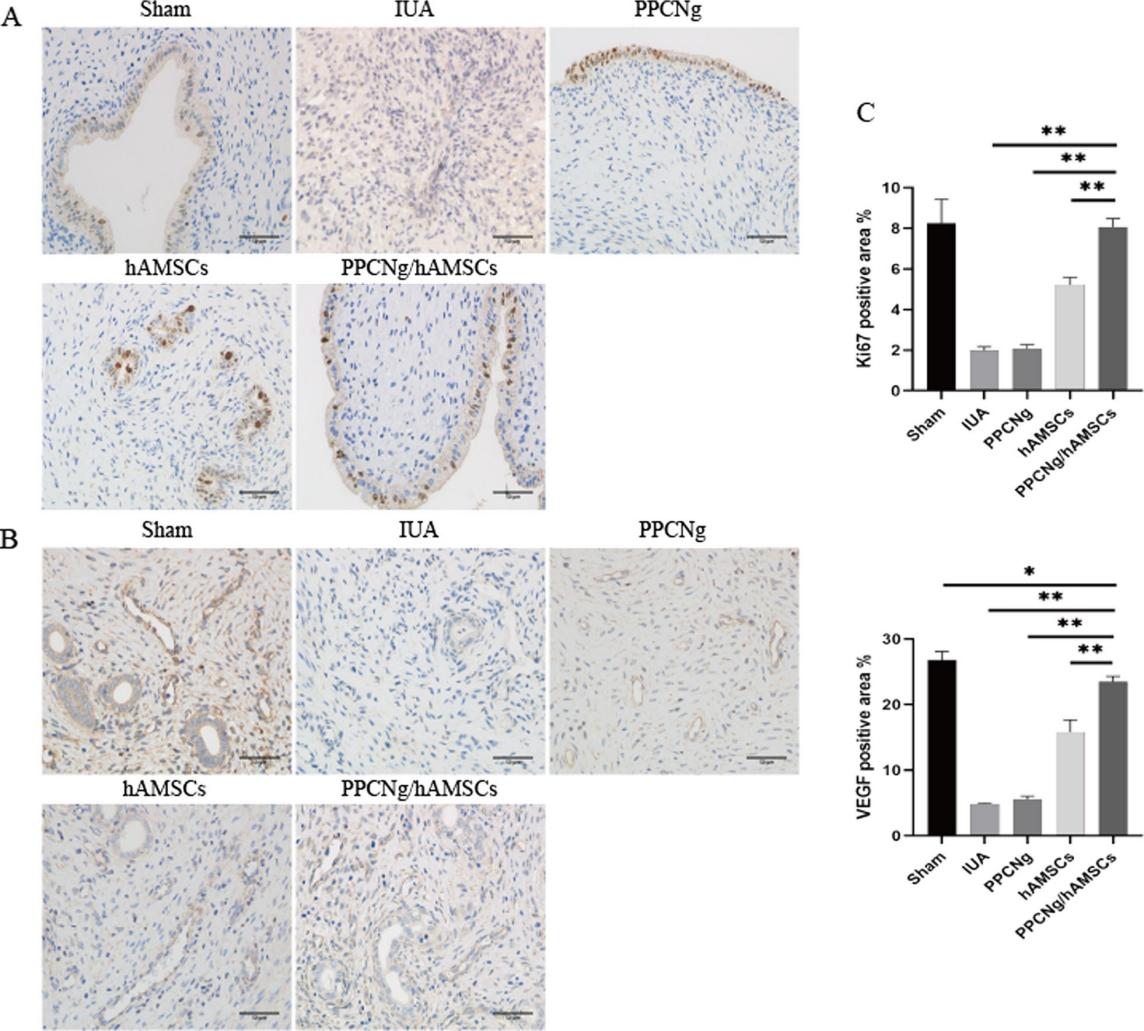
Cell proliferation and angiogenesis of endometrium. **A** Immunohistochemical staining of Ki67 (Scale bar = 50 μm). **B** Immunohistochemical staining of VEGF (Scale bar = 50 μm). **C** Statistical analysis of the percentage of Ki67, VEGF positive areas. (*n* = 3, *P < 0.05, **P < 0.01)

Ki67 is a nuclear transcription factor that is related to cell proliferation ability. Immunohistochemical results showed that Ki67 was mainly expressed in the nucleus of epithelial cells. The expression in the PPCNg/hAMSCs group (7.62 ± 0.55%) was significantly higher than that in the hAMSCs group (5.21 ± 0.37%) (*P* < 0.05) and the PPCNg group (2.08 ± 0.20%**)** (*P* < 0.01) (Fig. 8A).

Vascular endothelial growth factor (VEGF) can regulate angiogenesis, repair damaged tissue, and promote functional remodeling. Immunohistochemical results showed that the expression of VEGF in PPCNg/hAMSCs group (23.47 ± 0.83%) was significantly higher than that in the hAMSCs group (15.80 ± 1.84%) (*P* < 0.01) and the PPCNg group (5.53 ± 0.50%) (*P* < 0.01) (Fig. 8B).

**Fig. 9:**
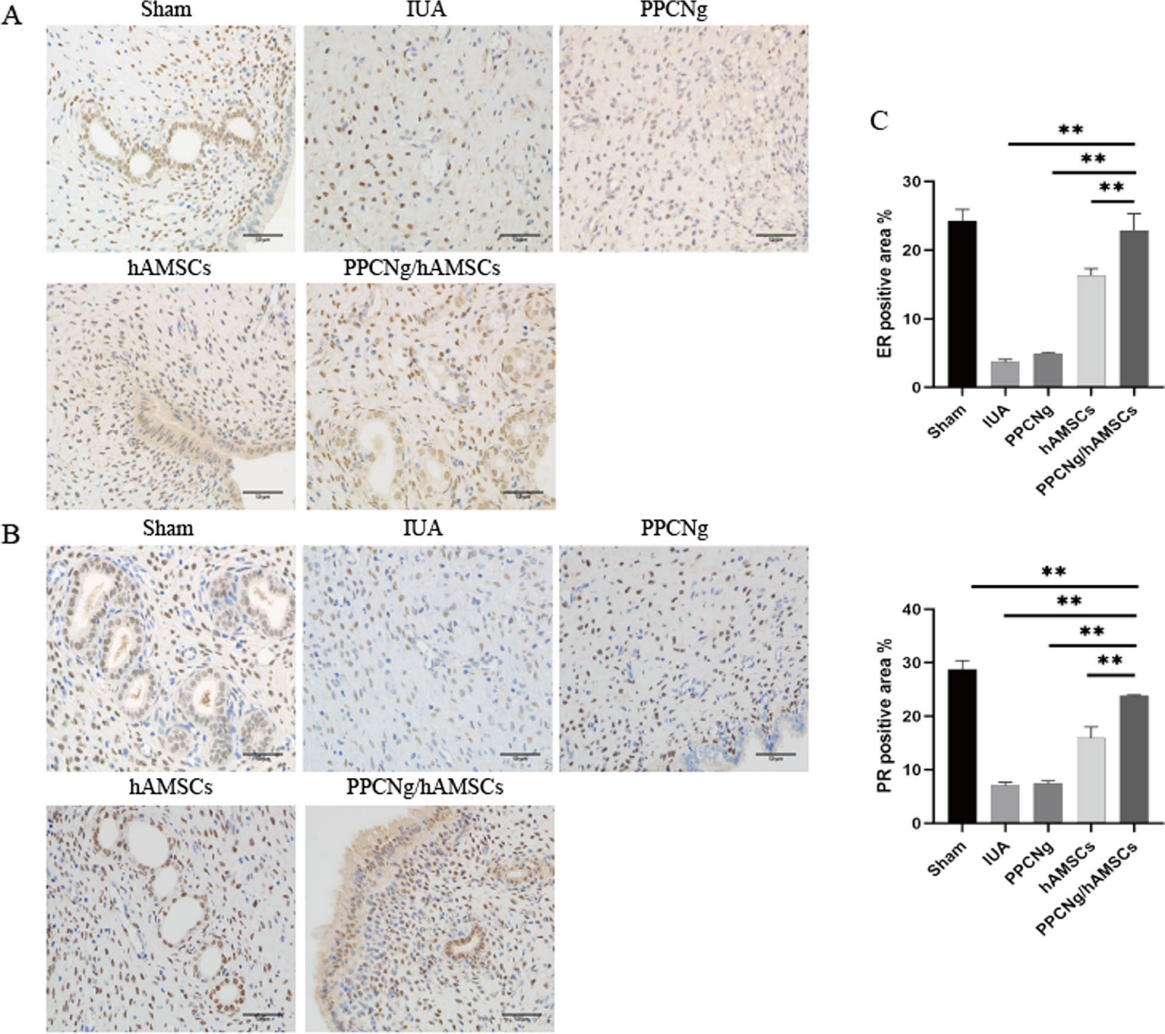
Endometrial receptivity recovery. **A** Immunohistochemical staining of ER (Scale bar = 50 μm). **B** Immunohistochemical staining of PR (Scale bar = 50 μm). **C** Statistical analysis of the percentage of ER, PR positive areas. (*n* = 3, *P < 0.05, **P < 0.01)

ER and PR are associated with endometrial receptivity. The results showed that ER and PR were mainly located in the nuclei of epithelial cells and mesenchymal cells. The expression level of both ER and PR in the PPCNg/hAMSCs group (22.89 ± 2.44%, 23.84 ± 0.14%) was significantly higher than that in the hAMSCs group (16.29 ± 1.02%, 15.96 ± 2.06%) (*P* < 0.01) and the PPCNg group (5.16 ± 0.41%, 7.46 ± 0.44%) (*P* < 0.01) (Fig. 9A, B).

### Restoration of reproductive function

**Fig. 10:**
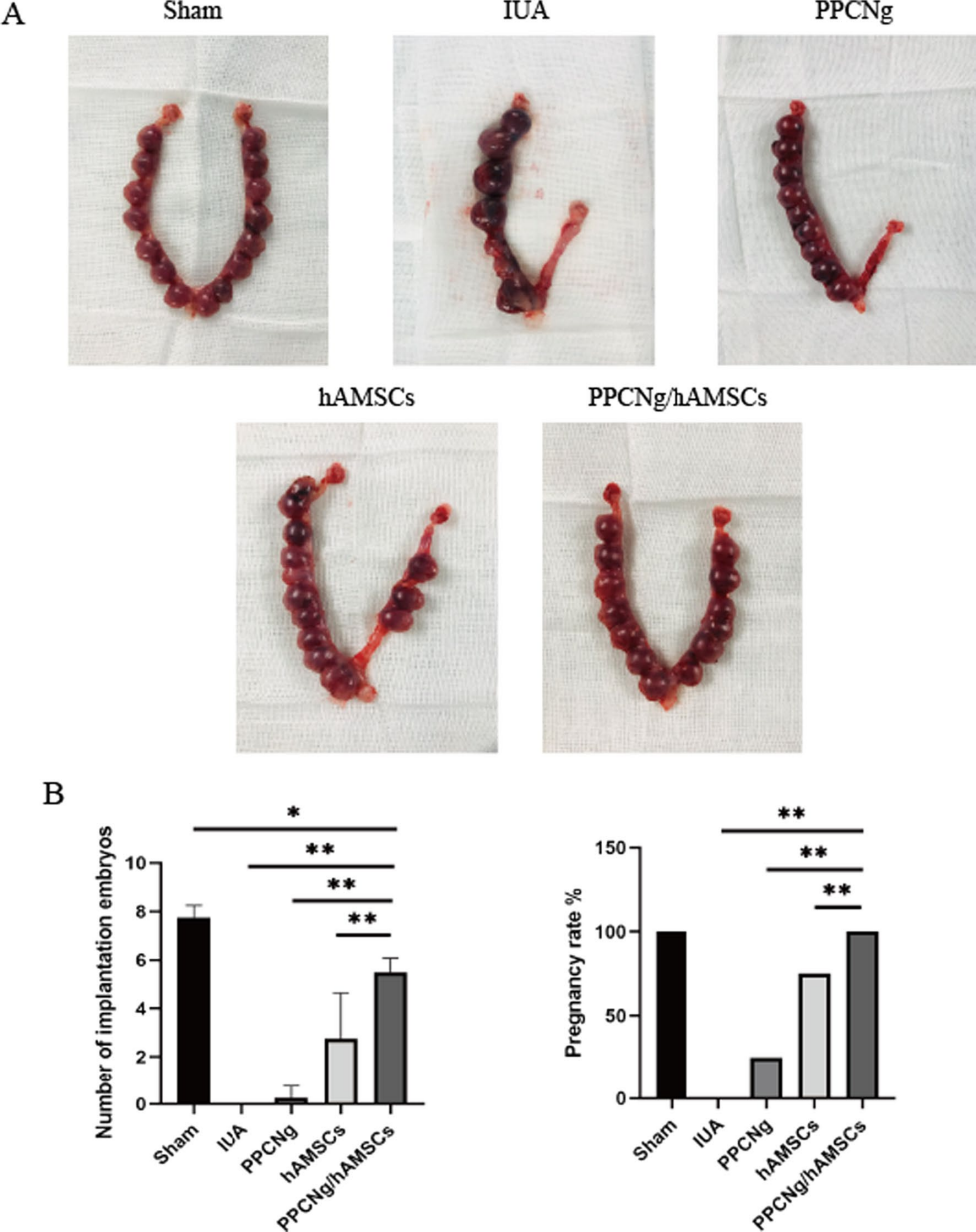
Fertility restoration. **A** The appearance of pregnant uteri. **B** Statistical analysis of the implantation embryo numbers and the pregnancy rate. (*n* = 4, *P < 0.05, **P < 0.01)

The function of the reconstructed endometrium was confirmed by evaluating reproductive capacity. At 14 d after gestation, no embryo implantation was observed in the IUA group. The pregnancy rate in the Sham group, the PPCNg group, the hAMSCs group and the PPCNg/hAMSCs group was 100%, 25%, 75% and 100%, respectively. Although the number of implantation embryos in the PPCNg/hAMSCs group (5.5 ± 0.6) was lower than that in the Sham group (7.75 ± 0.5) (*P* < 0.05), it was significantly higher than that in the hAMSCs group (2.75 ± 1.9) (*P* < 0.01) and the PPCNg group (0.25 ± 0.5) (*P* < 0.01) (Fig. 10B). These results demonstrated that hAMSCs combined with PPCNg intrauterine injection could promote fertility restoration effectively.

## Discussion

The endometrium consists of the functional layer and basal layer. The functional layer undergoes shedding and regeneration periodically under the regulation of steroid hormones, and the basal layer is responsible for the regeneration of the endometrium attributing to the native endometrial stem cells [38]. Severe endometrial injury in IUA results in the reduction or lack of endometrial stem cells and endometrial regeneration dysfunction, leading to endometrium thinning and decreased endometrial receptivity affecting embryo implantation [39, 40].

We have successfully conducted PKH26 labeled hAMSCs transplantation through intrauterine injection or tail vein injection in IUA rats, and found that hAMSCs could contribute to thick endometrium, abundant glands, relieving endometrial fibrosis, increasing the expression of epithelial markers and VEGF, and decreasing the expression of fibrosis-related factors; and found that the colonization rate of implanted hAMSCs in the injured uterus honors was not satisfactory via both transplantation approaches [41–43]. The poor in situ survival rate and utilization rate of cells may limit the therapeutic effect. Biocompatible scaffolds have been confirmed to improve the efficiency of transplanted cells [20, 44]. Intrauterine transplantation of MSCs mediated by appropriate biomaterials may contribute to the improvement of the prognosis of IUA.

PPCNg is a thermoresponsive mixture material possessing several biological characteristics such as biodegradability, intrinsic antioxidant properties and injectable properties, which has been demonstrated to support cells survival and proliferation in vivo after transplantation, ultimately promoting mature bone formation [29].

In this study, we explored the influence of PPCNg on hAMSCs. In vitro, hAMSCs were cultured in PPCNg to investigate the proliferation, apoptosis, phenotypic features and expression of pluripotency genes, as well as the inducing multilineage differentiation. The results showed that the proliferation and apoptosis rates of hAMSCs in PPCNg had no statistical difference from those of isolated-cultured hAMSCs, suggesting that PPCNg had no cytotoxicity. In addition, PPCNg had no significant effect on the phenotype expression of hAMSCs. There is a subpopulation of pluripotent very small embryonic-like stem cells (VSELs) among MSCs culture with the potential to differentiate into three embryonic germ layers [45, 46]. VSELs express several pluripotent markers, such as OCT4, SOX2, KLF4 and C-MYC [47]. These genes are crucial transcription factors for maintaining the pluripotency and undifferentiated state of embryonic stem cells, and their expressions are down-regulated after stem cells differentiation [48, 49]. MSCs show specific morphologies after differentiation, and differentiation-related genes play an important role in regulating and maintaining differentiation morphologies. In this study, the ability of cell differentiation was further explored by detecting the expression of genes related to differentiation. Cytokeratin is an epithelial-specific marker, and its expression is upregulated after differentiation into epithelial cells. RUNX2 is a key transcription factor for osteogenic differentiation [50], regulating the expression of ALP (early osteogenic marker) and BSP (late osteogenic marker), promoting osteogenic differentiation [51, 52]. PPARγ is the major inducer of adipogenesis, and LPL and ADIP are involved in lipid droplets formation and aggregation [53, 54]. SOX9, an early transcription factor for chondrogenic differentiation [55], regulates the expression of COL-II and ACAN, and plays a role in maintaining chondrogenic phenotypes [56]. We found that the expression levels of pluripotency genes and differentiation-related genes of hAMSCs cultured in PPCNg were not statistically different from those of isolated-cultured hAMSCs, suggesting that PPCNg did not affect the stemness and differentiation potential of hAMSCs. These results indicated that PPCNg has outstanding biocompatibility and can be applied to cell transplantation.

Immobilization and survival of cells in biomaterials is a necessary condition to improve the cell colonization rate. The hydrogel scaffolds are ideal cell carriers by entrapping cells and transporting them to the injured site, helping cells to colonize in situ and being highly permeable to oxygen, nutrients, and proteins [57]. PPCN shows a multi-layer microporous structure with a large number of pores, which can encapsulate cells and allow cell migration, while promoting the exchange of nutrients and metabolites through the pores, providing a favorable three-dimensional environment for cell growth. After in vivo transplantation, cells could be observed to migrate along the pore wall of PPCN [27]. In addition, the low syneresis of PPCN can prevent cells from mechanical force damage and protect cell vitality [27]. Compared with PPCN, PPCNg has better cell adhesion and shows higher cell survival rate [29]. In this study, we used PPCNg combined with DiR labeled hAMSCs for intrauterine injection transplantation, which has the advantages of easy operations and minimally invasion [58]. PPCNg formed gel in the uterine cavity of rats, where the temperature was about 38 °C, fixed cells, and completely adapted to the three-dimensional structure of the uterine cavity. Tracking implanted cells in vitro, the fluorescence intensity in the PPCNg/hAMSCs group was significantly stronger than that in the hAMSCs group, indicating that PPCNg increased the colonization rate of hAMSCs.

The degradation time of scaffolds needs to be consistent with the time of tissue regeneration to provide continuous structural and functional support throughout the regeneration process [59]. Previous studies have shown that PPCNg gradually begins to degrade at 3 weeks after subcutaneous injection and is completely absorbed in 4–5 weeks, and the degradation products are nontoxic [29]. In this study, the histological results showed that 2 weeks after transplantation, the endometrium structure in the PPCNg/hAMSCs group was significantly improved compared with the hAMSCs group, including thicker endometrium, more glands, and smaller fibrosis area, suggesting that the tissue repair began within 2 weeks after transplantation, which was shorter than the degradation time of PPCNg in vivo. These results suggested that PPCNg can encapsulate cells at the early stage of cell transplantation, providing a suitable growth environment for cells, improving the survival rate and utilization rate of hAMSCs, and being responsible for the recovery of endometrial structure.

In the treatment of IUA by hAMSCs, various cytokines participate in endometrial repair, improving the intrauterine environment, and recovering endometrial function. CK is a specific marker of epithelial cells and is related to cell differentiation [60]. The initial stage of endometrial repair is the regeneration of epithelial cells and covering the endometrial surface. In this study, the expression of CK7 and CK19 in the PPCNg/hAMSCs group was significantly higher than that in the hAMSCs group, indicating that PPCNg/hAMSCs can promote re-epithelization and form new endometrium. Ki-67 is a nuclear antigen, which is closely associated with cell mitosis and proliferation, mainly found in proliferating cells [61]. In the current study, the expression of Ki-67 was significantly increased in the PPCNg/hAMSCs group and was mainly expressed in endometrial epithelial cells, suggesting that PPCNg/hAMSCs promoted epithelial cell proliferation and contributed to the repair of endometrial epithelium. VEGF is the most important regulator of vascular growth, stimulating endothelial cell proliferation and promoting angiogenesis [62], resulting in increased nutrients, oxygen and hormones at the injured site [63]. The endometrial vessels closed and presented hypoxia in IUA, and the expression of VEGF and vascular density increased in endometrium post-therapy, revealing that angiogenesis plays an important role in endometrial repair [64]. In our study, the expression of VEGF in the PPCNg/hAMSCs group was significantly higher than that in the hAMSCs group, indicating that PPCNg/hAMSCs stimulated angiogenesis and provided nutritional supply for endometrial cell regeneration.

Restoring fertility is the ultimate goal of treatment for IUA, and endometrial function is assessed through embryo implantation ability. Embryo implantation is a complex process involving the embryo and endometrium, including embryo quality, endometrium receptivity and embryo-endometrium developmental coordination [65, 66]. In the treatment of IUA, endometrial receptivity is the key factor in evaluating the prognosis of fertility. A clinical study confirmed that endometrial thickness could be used to predict endometrial receptivity [67]. Zhang et al. [68] reported in a retrospective study that the highest rate of live births occurred when the endometrial thickness was at 8.7–14.5 mm, and endometrial thickness would affect implantation rate, pregnancy rate and live birth rate. We found that PPCNg/hAMSCs contributed to thicker endometrium and more epithelial cells. In addition, ER and PR are indicators of endometrial receptivity. Estrogen binds to ER to regulate endometrial proliferation and reproductive capacity [69, 70]. Progesterone is essential in the process of embryo implantation, and interference with progesterone function can lead to abortion or infertility [71]. Recent studies have shown that the expression of ER in IUA endometrium is significantly reduced [72]. Wang et al. [73] transplanted BMSCs into IUA rats and found that the expressions of ER and PR were significantly upregulated after treatment. In the current study, ER and PR were expressed in endometrial epithelium and stroma. The expression levels of ER and PR in the PPCNg/hAMSCs group were significantly higher than those in the hAMSCs group, suggesting that PPCNg/hAMSCs can enhance endometrial sensitivity to estrogen and progesterone and improve endometrial receptivity, and then regulate reproductive activities. Chi et al. [74] reported that the combination of estrogen and aspirin post-operation could improve endometrial receptivity by promoting angiogenesis and increasing intrauterine blood flow. Embryo implantation and development require a complete vascular network [70]. VEGF can induce angiogenesis and vascular permeability and regulate villous angiogenesis in early pregnancy [75]. We found that the expression of VEGF in the PPCNg/hAMSCs group was significantly upregulated. These results indicated that PPCNg/hAMSCs could regenerate endometrium with excellent embryo implantation conditions and increase the chances of a successful pregnancy.

At gestation d14, the PPCNg/hAMSCs group showed a higher pregnancy rate than the hAMSCs group. Further analysis showed that the number of embryos in the PPCNg/hAMSCs group was significantly higher than that in the hAMSCs group, and implantation embryos were found at the site of endometrial injury, suggesting extensive regeneration of functional endometrium, which could accept embryonic attachment and support embryonic development.

The most important pathological characteristic of IUA is endometrial fibrosis caused by fibroblast activity [76, 77]. Inflammatory cytokines and fibrotic cytokines act synergistically during the formation of fibrosis [78]. It’s reported that MSCs have anti-fibrotic properties due to their ability to secrete anti-inflammatory and anti-fibrotic factors [79]. Activation of TGF-β1 promoted the development of fibrosis and the expression of TGF-β1 and Smad3 were significantly up-regulated in IUA endometrium [80]. MSCs transplantation could down-regulate the expression of TGF-β, increase the activity of MMP-9 and delay epithelial-to-mesenchymal transition (EMT), thereby reducing fibrosis [81, 82]. Studies have found that MSCs increase the anti-inflammatory cytokines, such as FGF-β, IL-10 and IL-6, and decrease the pro-inflammatory cytokines, such as TNF-α, and IL-1β [83, 84]. The NF-κB signaling pathway is generally thought to be involved in inflammation, responses by inducing inflammatory cytokines [85]. Salama et al. [86] found that BM-MSCs up-regulated the expression of PCNA and VEGF, down-regulated the expression of NF-κB and effectively repaired the injured endometrium in the rat IUA model. Ma et al. [87] reported that MenSCs transplantation could promote endometrial repair in patients with refractory IUA, which showed increased endometrial thickness, prolonged menstrual duration, and increased pregnancy rate. In a current clinical study, patients with unresponsive thin endometrium caused by IUA received UC-MSCs therapy. Endometrial thickness and micro-vessel density were increased in all patients. Four of the 17 patients conceived, and three of them gave birth to live babies [88]. MSCs have a therapeutic effect on clinical IUA, but the number of clinical studies and recruited patients was small. The developmental mechanism of IUA in animal models was consistent with that in clinic. Animal model studies have documented that MSCs could repair the fibrotic endometrium in vivo through paracrine effect and anti-fibrotic pathways. However, the animal studies have focused on “incipient IUA,” with limited follow-up time, and the recurrence rate after treatment has not been reported. In this study, PPCNg can improve the utilization rate of hAMSCs and thus enhancing the therapeutic effect of transplanted cells in IUA. Nevertheless, whether MSCs can effectively address “established IUA” and reduce the recurrence rate need further investigation.

## Conclusion

This study showed that PPCNg had outstanding biocompatibility in vitro with no cytotoxicity on the growth of hAMSCs, and no influence on the phenotype, pluripotency or differentiation ability of hAMSCs. PPCNg significantly improved the utilization rate of hAMSCs in order to promote therapeutic effect. Moreover, PPCNg combined with hAMSCs transplantation presented exciting reproductive function restoration by promoting endometrial morphological recovery, stimulating endometrial epithelial cell regeneration, increasing uterine blood flow and upregulating the expression of factors related to endometrial receptivity. hAMSCs combined with PPCNg transplantation is a promising strategy, which provides a reliable alternative for the clinical treatment of IUA.

## Supplementary Information

**Additional file 1**. Human amniotic mesenchymal stem cells combined with PPCNg facilitate injured endometrial regeneration.

## Data Availability

The data that support the findings of this study are available from the corresponding author upon reasonable request.

## Abbreviations

IUA: Intrauterine adhesions
hAMSCs: Human amniotic mesenchymal stem cells
PPCN: Polyethylene glycol citrate-co-*N*-isopropylacrylamide
TCRA: Transcervical resection of adhesions
MSCs: Mesenchymal stem cells
OCT4: Octamer-binding transcription factor-4
SOX2: SRY-related high-mobility-group-box protein-2
KLF4: Kruppel-like factor-4
C-MYC: Cellular-myelocytomatosis viral oncogene
CK-7: Cytokeratin 7
CK-8: Cytokeratin 8
CK-19: Cytokeratin 19
E-CDA: *E*-Cadherin
ALP: Alkaline phosphatase
RUNX2: Runt-related transcription factor-2
BSP: Bone sialoprotein
SOX9: SRY-BOX transcription factor 9
ACAN: Aggrecan
COL-II: Type II collagen
PPARγ: Peroxisome proliferator-activated receptor *γ*
LPL: Lipoprotein lipase
ADIP: Adiponectin
