# Supplementary material for: Human amniotic mesenchymal stem cells combined with PPCNg facilitate injured endometrial regeneration
Source: Stem Cell Res Ther. 2022 Jan 12;13:17. doi: 10.1186/s13287-021-02682-2 (PMC8756707; doi:10.1186/s13287-021-02682-2)
Supplement: Supplementary file 1 — Additional file 1. Human amniotic mesenchymal stem cells combined with PPCNg facilitate injured endometrial regeneration. [file 13287_2021_2682_MOESM1_ESM.docx]

**Methods**

**Isolation, culture and Identification of hAMSCs**

Human placentas were obtained from healthy mothers tested negative for infectious diseases during full-term caesarean sections from the Obstetrics Department of the First Affiliated Hospital of Chongqing Medical University. hAMSCs were isolated and cultured according to our previous protocol [1]. The amniotic membranes were mechanically separated from the placentas and were cut into pieces. The amnion pieces, followed by twice digestion with 0.05% trypsin at 37°C for 30 min and once with 0.1% collagenase II (Meilunbio) at 37°C for 1 h. The remaining tissue pieces were filtered and centrifuged. Isolated hAMSCs were cultured in Dulbecco’s Modified Eagle’s Medium: F12 (DMEM/ F12) (Gibco) supplemented with 10% fetal bovine serum (FBS) (PAN, Germany), 1% penicillin and streptomycin (Beyotime) and incubated at 37°C in 5% CO2. The culture medium was changed every 2 to 3 days. When cells reached 80%–90% confluence, adherent cells were passaged via digestion with 0.25% trypsin. hAMSCs in the third passage were used for subsequent experiments. The cells were identified by the expression of specific surface markers. Cells were incubated with the antibodies of anti-CD44 (ab189524, Abcam), anti-CD105 (ab231774, Abcam), anti-CD34 (ab81289, Abcam) and anti-CD45 (ab40763, Abcam) for 15 min, stained with the Alexa Fluor 488-conjugated secondary antibody (ab150077, Abcam) for 10 min, and finally assessed by flow cytometry (FACSCalibur, Bio-Rad).

**Results**

**Morphology and surface markers of hAMSCs**

The isolated hAMSCs grew adherently in a vortex-like arrangement and showed a fibroblast-like morphology with a spindle-shaped exterior (Fig. S1A). Flow cytometry results revealed that hAMSCs highly expressed mesenchymal stem cells surface markers, such as CD44 (99.32%) and CD105 (99.83%), but did not express hematopoietic markers, including CD34(0.61%) and CD45(0.16%), confirming that the cells were target hAMSCs (Fig. S1B).


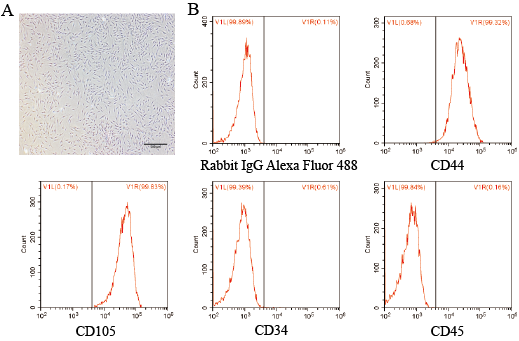


**Fig. S1** hAMSCs identification. A) The morphology of hAMSCs. B) Detection of CD44, CD105, CD34, CD45 by flow cytometry.

**References**

1. Gou Y, Zhang W, Li C, Yu J, Mao Y, Wu B, et al. Role of NF-κB signaling pathway in treatment of intrauterine adhesions by human amniotic mesenchymal stem cells. Acta Acad Med Mil Tert. 2020;42(11):1101-8. [https://dx.doi.org/10.16016/j.1000-5404.202003065.](https://dx.doi.org/10.16016/j.1000-5404.202003065)
